# Stability of Optical and Mechanical Properties in Multi-Shade, Group-Shade, and Single-Shade Resin Composites

**DOI:** 10.1155/ijod/3647128

**Published:** 2025-06-23

**Authors:** Violina Gunawan, Joanne Jung Eun Choi

**Affiliations:** Sir John Walsh Research Institute, Faculty of Dentistry, University of Otago, 310 Great King Street, Dunedin 9016, New Zealand

**Keywords:** mechanical properties, optical properties, resin composite, restorative, single-shade, universal-shade

## Abstract

**Objectives:** To investigate the effect of various immersion media on the optical, mechanical, and surface properties of multi-shade (MS), group-shade (GS), and single-shade (SS) dental resin composite (RC).

**Materials and Methods:** Three different RCs (MS, GS, and SS) were involved in this study (*n* = 8/group). Rectangular specimens (10 mm × 10 mm × 2 mm) were fabricated and thermocycled in artificial saliva, black coffee, and red wine for 10,000 cycles. Color stability (∆E_00_), relative translucency parameter (RTP), surface roughness (Sa), and Vickers hardness (VH) were measured. Data were statistically analyzed (SPSS v27) and surfaces analyzed under scanning electron microscopy.

**Results:** All ∆E_00_ values exceeded the acceptability and perceptibility threshold (PT). All RC exhibited statistically significant changes in RTP, Sa, and VH after 10,000 thermal cycling (*p*  < 0.001). There are statistically significant changes of ∆E_00_ and translucency for specimens immersed in red wine (*p*  < 0.001). No immersion media exert statistically significant changes on Sa (*p* = 0.004) and hardness (*p*  < 0.086).

**Conclusions:** SS RC shows the best stability in both optical and mechanical properties, whereas GS RC exhibits variable performance with no trend requiring further investigations. The MS RC shows the least color and translucency stability but highest hardness throughout the experiment.

## 1. Introduction

Dental resin composite (RC) is a commonly used dental direct restorative material due to its properties [1–4]. Utilizing chemical and micromechanical bonding, it allows conservative tooth preparation. It has high polishability, can be translucent and esthetic. On the other hand, human perception on teeth color is very complex, as enamel and dentin are layers with different opacity and translucency [2, 5]. To mimic the polychromatic nature of dentition, a variety of multiple shaded composites were manufactured, as RC has low biomimetic potential due to their noncrystalline structures. It relies on dyes in their contents to produce different opacities and translucencies based on Vita Classical Shade Guide [1, 5]. Additionally, every tooth has different shades depending on sites, morphology, and age. This makes color matching between natural dentition and RC very complicated as layering technique is required to achieve maximum esthetic results [1, 6, 7], leading to increased RC inventory costs, chair-time, unused composite shades, and reliance on shade-matching procedure [5].

Recently, Spectra ST (a group-shade [GS] RC) and Omnichroma (a single-shade [SS] RC) were marketed to overcome the issues mentioned above. Spectra ST was marketed to simplify the shade-matching process, where one color of Spectra ST suits multiple shades of teeth on Vita Classical Shade Guide [8]. This is possible due to its SphereTEC fillers with prepolymerised fillers that allows blending effect, or more commonly known as “chameleon” effect. Previous study found that Spectra ST has comparable shade-matching ability to the conventional multi-shade (MS) RC, such as Estelite Sigma Quick [9]. On the other hand, Omnichroma is marketed to eliminate the shade-matching process altogether, where one color fits all different shades of teeth on Vita Classical Shade Guide. It utilizes structural color based on 260 nm spherical fillers that reflects visible light wavelengths of red to yellow range, enabling them to blend with the surrounding tooth structure [2]. Albeit promising, there is limited evidence on its success on shade matching ability in simulated dental restorations [2, 7, 9].

Despite the advantages, all restorative materials are subjected to degradation once it is exposed to the oral environment for a long period of time. Whether it is staining, recurrent decay, discoloration, and loss of gloss, these variables affect RC longevity negatively [10]. Hence, the modified United States Public Health Services (USPHS) criteria was proposed as a systematic assessment of dental restorations clinically [11]. The criteria expand on the evaluation of esthetic, functional, as well as biological properties of RC, including color match and translucency, surface luster, material fracture, and retention. Superficial staining has been reported as one of the main failures of CR, indicating restoration replacement [2, 4, 12]. The discoloration may occur by intrinsic factors, but most likely extrinsic factors, such as absorption of pigments from food and drinks [4].

Several papers have investigated how the aging process influences the properties of RC. The conflicting results may be a consequence of different methodologies. One study concluded that MS RC (Filtek Z-350) has lower color stability (∆E_00_) compared to SS RC (Omnichroma) after immersed in room temperature coffee for 2 weeks [2]. Another study found no significant difference in ∆E_00_ of MS RC (Filtek Z-350, Estelite Palfique LX5, and IPS Empress Direct) and SS RC (Omnichroma) [3], while [4] found that SS RC is prone to unacceptable color changes and Sa after aging. However, there is yet literature that investigated the stability of GS and SS RC properties under thermocycling environment, especially on their mechanical properties. Therefore, the primary aim of this study was to analyze the stability of optical and mechanical properties of three different RC in a thermocycling environment. The optical properties measured were change in color and translucency parameter, whereas the mechanical properties tested were Sa and Vicker's hardness (VH). The null hypotheses of this study were that artificial aging with different immersion media has no influence on (1) ∆E_00_, (2) translucency parameter, (3)Sa, and (4) hardness of GS RC and SS RC, compared to MS RC.

## 2. Materials and Methods

A sample size calculation was performed by G^*∗*^power v3.0.10 software (Heinrich-Heine-Universtät Düsseldorf), referring to previous study with similar methodology [2]. The calculation revealed that a minimum of eight samples in each category was required to achieve a 95% confidence interval. Hence, a total of 72 specimens (*n* = 24/group) from three different types of RC were prepared for the study.

### 2.1. Specimen Preparation

Three different types of resin composites (RCs) were used in this study (Table 1). The specimens were fabricated in size of 10 mm × 10 mm × 2 mm to ensure adequate surface area and thickness for testing. The dimension was selected to be big enough for the tip of the light curing machine and color spectrometer used [13, 14]. The RCs were packed into 3D-printed silicone mold (PolyFlex TPU95 printed in Prusa MKi3) and cured according to manufacturer instructions using curing light with intensity of 1100 mW/cm2 (Bluephase LED Cure Light). The specimens were inspected macroscopically for any defect prior to being included in the study. Then, four different levels of grit (320, 500, 1200, and 2400-grit) silicon carbide paper were used on 230 rpm (TegraPol-21, Struers, Denmark) to polish the specimens. Their Sa was measured and ensured to be under 0.2 µm as it is the clinically acceptable threshold for Sa [15]. All specimens were submerged in reversed osmosis water for 24 h prior to baseline measurements of optical and mechanical properties. All measurements were done by a single operator for standardization effort.

### 2.2. Thermocycling

Each specimen was immersed in the containers with 2.5 mL of either artificial saliva, black coffee, or red wine (Table 2). Then, the specimens were thermocycled for 5000 and 10,000 cycles to simulate 6-months and 12-months in vivo respectively, alternating between 5°C and 55°C water bath with dwell time of 15 s, which equates to 1 year of clinical services as per previous studies protocols [16, 17]. The solutions were changed between each subgroup measurements for consistency and premade can coffee was used for consistency instead of manually preparing the granulated coffee in a specific ratio as listed in Table 2.

#### 2.2.1. Measurement of Change in Color (∆E)

The specimens were patted dry with paper towel prior to color measurements. The digital spectrophotometer (Vita Easyshade V, Zahnfabrik, Germany) was used and calibrated according to manufacturer's instructions prior to each measurement. All color measurements were conducted when specimens were placed on a standard white background paper (Leneta Form 2A), in accordance with the ISO/TR Z8642:Z016(E) inside a photo box to have the consistent lighting. Each specimen was measured three times and the values are averaged before being inputted into the CIEDE2000 formula [18–21]. The CIEDE2000 formula is the following:

  $$\begin{array}{c} \Delta {\text{E}}_{00}=\;\sqrt[{2}]{{\left(\frac{\Delta L^{\prime }}{k_{L}S_{L}}\right)}^{2}+{\left(\frac{\Delta C^{\prime }}{k_{C}S_{C}}\right)}^{2}+\;{\left(\frac{\Delta H^{\prime }}{k_{H}S_{H}}\right)}^{2}+R_{T}\left(\frac{\Delta C^{\prime }}{k_{C}S_{C}}\right)\left(\frac{\Delta H^{\prime }}{k_{H}S_{H}}\right)}, \end{array}$$

where ∆E_00_ is the color difference, ∆*L*, ∆*C*, and ∆*H* are the difference in lightness, chroma, and hue respectively, and RT is the rotation factor that considers the interactions among hue and chroma differences in the blue area. Weighting functions SL, SC, and SH are modulated the total color difference for variation in the site of the color difference pair in the L^*∗*^, a^*∗*^, and b^*∗*^ coordinates, and the parametric factors KL, KC, and KH were the expressions for the experimental conditions. These are empirical terms for converting the differences for each coordinate into the CIEDE2000 difference formula. The CIEDE2000 parametric factors were adjusted to 1.

### 2.3. Measurement of Relative Translucency Parameter (RTP)

The specimens were measured using a spectrophotometer, three times each on black and white background paper (Leneta Form 2A), in accordance with ISO/TR Z8642:Z016(E). Generally, translucency parameter measurement requires an optically uniform material throughout the thickness of the material. However, specimens that were thermocycled in coffee solution results in nonuniform staining. Therefore, the RTP technique was used instead [22]. The formula of RTP is as follows:

  $$\begin{array}{c} {\text{RTP}}_{\text{CIEDE}00}=\;\sqrt[{2}]{{\left(\frac{\Delta L^{\prime }}{k_{L}S_{L}}\right)}^{2}+{\left(\frac{\Delta C^{\prime }}{k_{C}S_{C}}\right)}^{2}+\;{\left(\frac{\Delta H^{\prime }}{k_{H}S_{H}}\right)}^{2}+R_{T}\left(\frac{\Delta C^{\prime }}{k_{C}S_{C}}\right)\left(\frac{\Delta H^{\prime }}{k_{H}S_{H}}\right)}. \end{array}$$

### 2.4. Measurement of Sa

Sa was measured in a noncontact 3D Sa and optical profiler (TopMap Micro.View, Polytec) using a 10x objective lens. Four frames of the surface area were captured and stitched together to yield an average Sa value in micrometres (μm). The Sa over the roughness threshold (Sa = 0.2 μm) causes a simultaneous increase in biofilm accumulation, and no further decrease in bacterial adhesion could be observed under the threshold value [15], and smooth surfaces add to the comfort of the patient as a Sa change of 0.3 µm can be identified by the tip of the tongue [23].

### 2.5. Measurement and Calculation of VH

The polished specimens were indented using Universal Testing Machine (Instron 3369) with diamond indenter at load of 50 N with loading time of 10 s. Three indentations were made on each specimen at each time point and the length of the indentation was inspected under microscope (Nikon DS-Fi2) with 6.3x magnification. The ruler was calibrated and used to measure the length of the indentation, which becomes the data that is used to calculate VH of the specimens with the following formula:

  $$\begin{array}{c} \text{VH}=\;1.854\left(\frac{F}{D^{2}}\right) \end{array}$$

when, *F* is the applied load (measured in kilograms-force) and *D*^2^ is the area of the indentation (measured in square millimeters).

### 2.6. Scanning Electron Microscope (SEM) Imaging and Backscatter Electron (BSE) Imaging

Specimens were prepared for observation under the SEM by mounting each one on aluminum stubs, using double-sided carbon tape. Each specimen was then coated with approximately 10 nm of carbon in a Peltier-cooled high-resolution sputter coater (Emitech K575X; EM Technologies Ltd.) fitted with a carbon coater (Emitech 250X; EM Technologies Ltd.). Analysis was conducted by field emission SEM (JSM-6700F; JEOL) and magnification range of 100–15,000. Imaging was done at an accelerating voltage of 10 kV and a chamber pressure of 10^−4^ Pa.

### 2.7. Statistical Analysis

The normality test and statistical analysis was conducted using IBM SPSS Software (version 28). Kolmogorov–Smirnov normality test was done for groups of immersion liquid dataset, and Shapiro–Wilk normality tests were conducted to analyze the dataset based on thermocycling dataset. The statistical difference between groups were analyzed using one-way ANOVA followed with Bonferroni post hoc test for parametric dataset, whereas independent samples Kruskal–Wallis test followed with Pairwise Comparison post hoc test were used to analyze nonparametric ones. The Type 1 statistical error was performed at a preset alpha of 0.05.

## 3. Results

### 3.1. ∆E_00_

**Figure 1:**
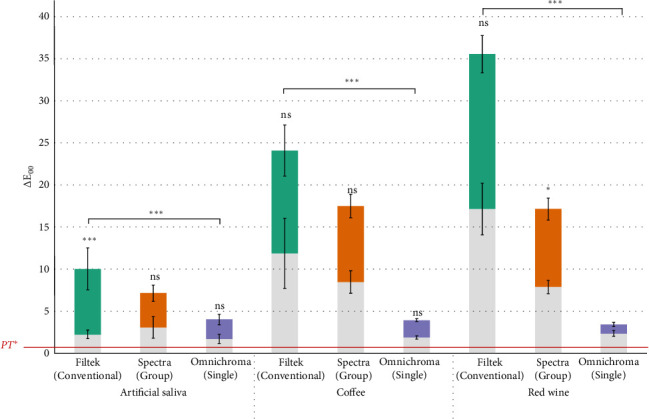
Bar graph showing the trends in color stability of three different types of RCs when immersed in three different medium and thermocycled. The top part of the each bar is T_1_–T_0_ and bottom part in gray is T_2_–T_1_. ‘ns' stands for no significant difference and *⁣*^*∗*^ represents when *p*-value is < 0.05 and *⁣*^*∗∗∗*^ represents *p*-value <0.001. The red line for PT^*∗*^ represent perceptibility threshold.

The ∆E_00_ of all specimens under different treatments were measured and calculated, as shown in Table 3 and Figure 1. The highest change in color was found in MS specimens irrespective of the immersion liquid, followed by GS and SS specimens. There was no statistically significant difference between MS and GS when immersed in artificial saliva (*p* = 0.167) and black coffee (*p* = 0.285). However, the difference between MS and GS was statistically significant in the red wine group (*p* < 0.001). On the other hand, SS specimens had statistically significant differences when compared to MS groups (*p* < 0.001).

### 3.2. RTPs (CIEDE 2000)

**Figure 2:**
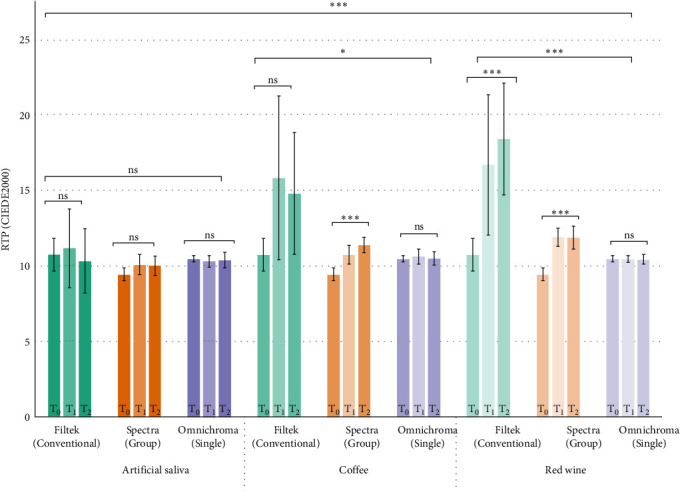
Bar graph showing the trends in relative translucency parameter stability of three different types of RCs when immersed in three different medium and thermocycled. ‘ns' stands for no significant difference and *⁣*^*∗*^ represents when *p*-value is < 0.05 and *⁣*^*∗∗∗*^ represents *p*-value < 0.001.

The RTP values are presented in Table 4 and Figure 2. After 10,000 thermocycles, the highest translucency was found in SS specimens irrespective of the immersion liquid. Both GS and SS specimens showed statistically significant differences to MS specimens within the red wine group only (*p*  < 0.001).

### 3.3. Sa

**Figure 3:**
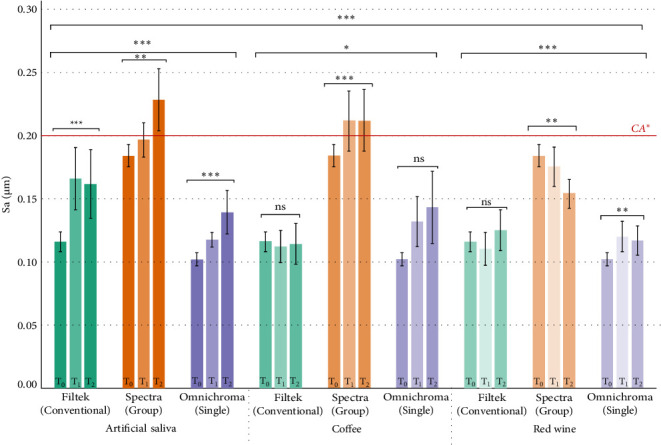
Bar graph showing the trends in surface roughness of three different types of RCs when immersed in three different medium and thermocycled. ‘ns' stands for no significant difference and *⁣*^*∗*^ represents when *p* value is < 0.05, *⁣*^*∗∗*^ represents *p*-value < 0.01 and *⁣*^*∗∗∗*^ represents *p*-value < 0.001. The red line for CA^*∗*^ represents clinically acceptable threshold.

The Sa values are presented in Table 5 and Figure 3. After 10,000 thermocycles, the highest Sa was found in GS group, followed by MS and SS groups respectively. There were no statistically significant differences between MS and SS groups across the different immersion liquid groups (*p*  > 0.05). On the other hand, there were statistically significant differences between MS and GS groups in artificial saliva and black coffee groups (*p* < 0.001). Only GS specimens treated in artificial saliva and black coffee exceed the recommended Sa threshold of 0.2 µm at the end of 10,000 cycles.

### 3.4. VH

**Figure 4:**
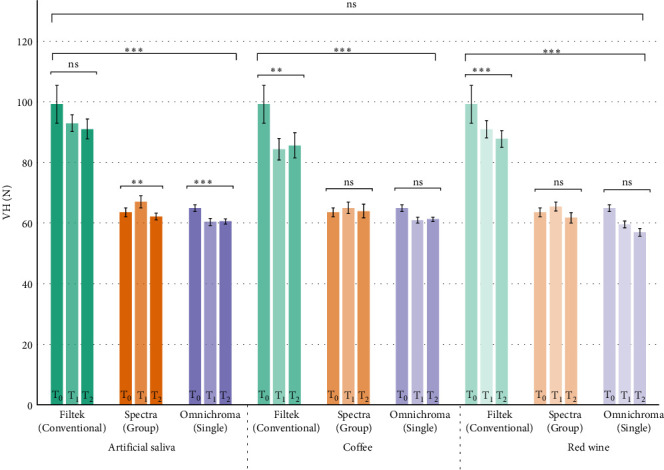
Bar graph showing the trends in hardness of three different types of RCs when immersed in three different medium and thermocycled. ‘ns' stands for no significant difference and *⁣*^*∗*^ represents when *p*-value is < 0.05, *⁣*^*∗∗*^ represents *p*-value < 0.01 and *⁣*^*∗∗∗*^ represents *p*-value < 0.001.

Overall, the highest hardness measured after 10,000 cycles was found in MS specimens, followed by GS and SS specimens respectively (Figure 4 and Table 6). The general trend showed a decrease in Vickers hardness after 10,000 cycles from baseline measurements. All GS and SS specimens showed statistically significant differences compared to MS specimens in artificial saliva, black coffee, and red wine (*p*  < 0.001).

### 3.5. SEM and BSE Imaging Analysis

**Figure 5:**
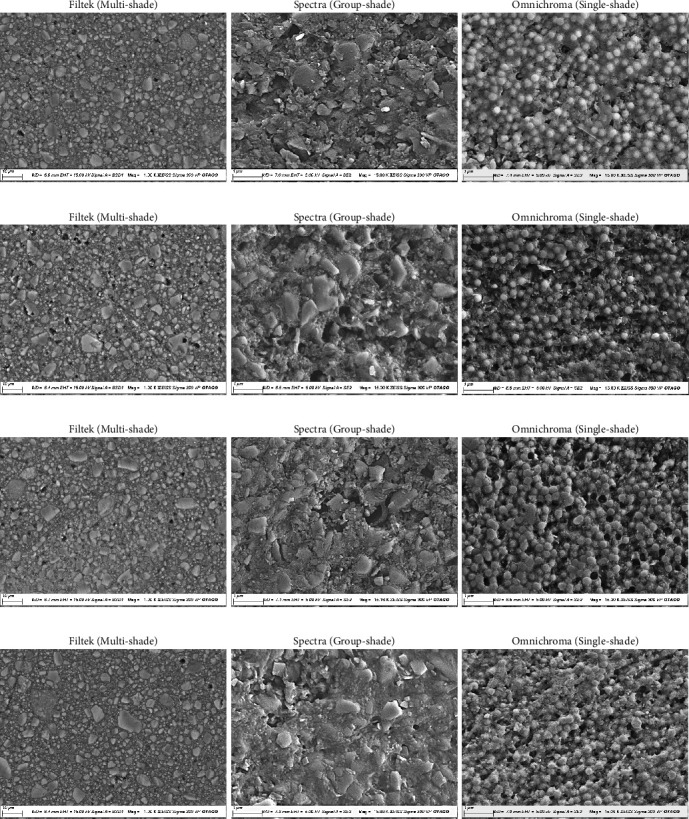
SEM images showing surface topography of three different types of RCs at baseline and compared to 10,000 thermocycles in three different immersion liquids. (a) baseline, (b) immersed in artificial saliva, (c) immersed in black coffee, and (d) immersed in red wine.

The SEM and BSE Imaging Analysis was done to visualize the topography of the specimens (Figure 5). Generally, all specimens showed markings, filler fall-outs or cluster or debonding and pre-polymerized fillers (PPF) in the RC. MS specimen fillers were observed under BSE imaging and appeared to be geometrically irregular with various sizes. Similar to MS and GS under SEM imaging shows circular but irregular fillers. SS specimens showed uniformly distanced fillers with same shape and sizes.

## 4. Discussion

The first null hypothesis of the study was that artificial aging with different immersion media has no influence on ∆E_00_ of GS and SS RC when compared to MS. The findings of this study partially reject the first null hypothesis. The key explanation to the optical behavior of RCs lies on the filler and resins. Stain penetrates into RC via microcrack development due to the degradation of resin-filler interface [3]. Microcrack development is caused by hygroscopic expansion during thermocycling [24]. Additionally, the combination of thermal and hydrolytic degradation from repeated sudden temperature changes. The high temperature gradient differences in thermal expansion coefficient between resin matrix and filler particles also causes repetitive shrinkage and expansion, resulting in different volumetric changes between resin and filler particles, leading to formation of microfractures in the resin-filler interface [4, 10]. The hydrophilicity of the resins also influences water sorption, thus stain penetration.

When compared to MS RC, GS RC contains PPF that lack active binding sites for surface coupling agents, leading to reduced bonding to the resin matrix [24]. SS RC utilizes structural color technology known to be less prone to change in color over time due to reduced photochemical degradation and less color distortion [6]. Furthermore, SS RC higher hydrophobic resins (UDMA and TEGDMA), accounts for its statistically significant lower color change, compared to MS and GS RCs that contains hydrophilic bis-GMA.

According to [25] and 2019, the 50:50% perceptibility threshold (PT) and acceptability threshold (AT) for ΔE_00_ in dental materials are approximately 0.8 and 1.8, respectively. In our study, all RC groups exceeded both thresholds following thermocycling, indicating that the color changes were not only perceptible to the human eye but also clinically unacceptable. These findings underscore the influence of material composition and immersion media on long-term esthetic outcomes.

For RTP, only SS RC immersed in black coffee and red wine, and GS RC immersed in red wine presented statistically significant differences when compared to MS RC. Therefore, the second hypothesis of the study is partially rejected.

Translucency is affected by the light transmission, which is determined by the difference in refractive index between the filler and resin. The smaller the difference, the higher the translucency of the material ([7] b). Higher filler content also directly improves translucency due to optimal optical scattering of the composite [1, 2], and SS RC has the highest filler volume percentage compared to MS and GS RC.

Presence of bis-GMA can also increase translucency. Although SSRC doesn't contain bis-GMA, it still shows the best translucency stability compared to MS and GS RC. This is most likely attributable to its specific combination of filler size and fraction that match the resin.

**Figure 6:**
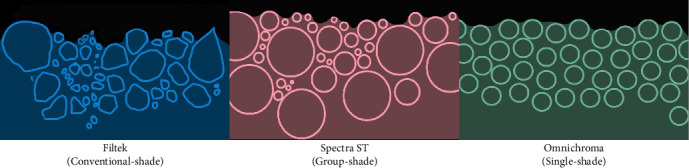
Image illustrating the cross-sectional view of the filler particles in the RCs studied.

The third null hypothesis of the study was that artificial aging with different immersion media has no influence on Sa of GS and SS RC when compared to MS. The study found that only GS RCs have statistically significant differences compared to MS. Therefore, the third hypothesis is partially rejected. Filler characteristics, such as size, shape, and distribution—significantly influence Sa, discoloration susceptibility, and microhardness. The SEM and BSE images confirmed clear differences in filler morphology among the tested materials. MS specimens exhibited irregular and polydisperse filler particles, while GS composites displayed larger, irregularly shaped barium glass fillers. SS materials showed uniformly spaced spherical fillers as illustrated in Figure 6, which have been associated with more consistent light scattering and surface behavior. These morphological differences contribute directly to the observed variations in Sa and discoloration. Larger or irregular filler particles are more prone to plucking and interfacial debonding during mechanical and thermal stress, leading to increased Sa and discoloration [4, 24]. Moreover, fillers with high leachability, such as barium glass, are particularly susceptible to degradation in acidic or alcoholic environments, which contributes to both roughness and color instability [26, 27].The mechanical property differences, such as Vickers hardness, are also partly attributed to filler type and dispersion. For instance, materials with smaller, uniformly distributed fillers or reinforced with ZrO_2_ and SiO_2_ tend to show enhanced hardness and aging resistance [28–30]. This is consistent with our findings, where MS composites—containing aggregated Zr/Si clusters—exhibited the highest microhardness values across all immersion media.

Moreover, cured RCs initially have a smooth resin-rich surface that is removed upon polishing [24, 27, 31]. Therefore, the bigger the size of the fillers, the more irregular the surface of RC becomes and translates into higher Sa. Secondly, barium glass is an excellent radio-opacifying filler, but leaches away when in contact with acidic drinks, high-pH solutions and other oral fluids [27]. It also has higher leachability compared to silica fillers due to ionic exchange mechanism on the filler surface when stored in artificial saliva [26]. These explain why GS RC (which contains Barium glass fillers) suffers from higher Sa after artificial saliva, black coffee and red wine immersion. On the other hand, MS and SS have smaller filler size compared to GS RC, in which degradation of the fillers and/or resin properties will result in less Sa. Additionally, alcohol causes plasticizing effect which in turn causes softening of the resin [32], making it more leachable. Bis-GMA and UDMA is inherently more viscous and leachable, which leads to lower degree of conversion and polish retention [24, 27, 31], hence the higher Sa in MS and GS that contain bis-GMA. It is also note-worthy that higher Sa is also associated with increased staining [2].

Regarding the artificial aging with different immersion media has no influence on Sa of GS and SS RC when compared to MS, the final null hypothesis was rejected. The high leachability of bis-GMA and UDMA can also lead to lower hardness. Addition of TEGDMA will overcome this issue as it has lower molecular weight, and consequently increases degree of conversion, improves handling consistency as well as necessary filler incorporation [27]. A relative increase in filler size, along with irregular shape leads to superior mechanical properties, such as greater resistance to accelerated aging and higher reliability [28]. This explains why MS RC has highest VH amongst all three groups throughout all time points, considering it has irregular shaped fillers. Additionally, incorporation of ZrO_2_ results in high strength and low abrasion resin with desirable esthetic properties [29]. SiO_2_ also leads to significant increase in hardness [30].

The methodology of this study was constructed based on the existing literature while addressing previous limitations. When measuring ∆E_00_, the newly established CIEDE2000 formula has proven to be better and sophisticated compared to other formulas, as well as better in reflecting color perceived by human eyes [19]. On the other hand, non-contact 3D optical roughness measurement involves coherence scanning interferometry with a broadband white light source [33], which provides a 3D surface map without scratching the specimens and preserves their integrity [4]. Unlike contact stylus profilometers, it also allows controlled surface area selection, which allows standardization. Thermocycling has been commonly employed in dental research as the system conventionally simulates in-vivo aging of restorative materials. It recreates the thermal stress of the oral cavity via repeated cycling of hot and cold temperature water exposure. Although there is a lack of standardization protocol, this method is widely used and seems to be a valid in vitro method to simulate accelerated aging of restorative materials [16, 17]. This became the limitation of the study at the same time as it was logistically difficult to confirm whether each specimen reached the temperature of 5°C and 55°C. To further understand the aging behaviors of different RCs, future studies should investigate the influence of different thickness (i.e., restoration depth) of these RCs on their optical and mechanical properties and stability.

## 5. Conclusions

Within the limitations of this current study, it was concluded that:

1. RCs with different shade-matching abilities exhibit distinct behaviors in ∆E_00_, translucency, Sa, and hardness when subjected to simulated aging.
2. MS composites, while offering higher initial hardness are shown to be more prone to discoloration and surface changes over time. GS (Group/blended-shade) composites presented variable performance, indicating that clinicians should assess them carefully depending on clinical scenario. SS composites demonstrated the most consistent optical and mechanical stability, likely due to their uniform filler morphology and hydrophobic resin formulation. This makes them potentially more suitable in esthetically demanding cases, especially where shade matching may be difficult.
3. Thermocycling has statistically significant impact for all specimens after 10,000 cycles in RTP, Sa and VH. All specimens also exceeded the acceptability and PT. These findings highlight the importance of considering both optical and mechanical aging behavior when selecting restorative materials.

## Figures and Tables

**Table 1:** Composition of different composite resins used in the study.

CR group	CR brand	Manufacturer	Resin	Filler	Classification
Multi-shade	Filtek Supreme XTE Universal Restorative Syringes (A2 body shade)	3M, St. Paul, Minnesota, USA	Bis-GMA, UDMA, TEGDMA, bis-EMA, and PEGDMA	(78% wt and 63.3% vol) 20 nm SiO_2_, 4–11 nm ZrO_2_, and 0.6–10 µm aggregated Zr/Si clusters	Nanohybrid

Group-shade	Spectra ST High Viscosity (A2 cloud shade)	Dentsply Sirona, Charlotte, North Carolina, USA	Urethane-modified Bis-GMA and TEGDMA	(77.2% wt and 57% vol) 15 µm silanated glass particles of Ba, B, F, Al, SiO_2_, and TiO_2_ glass	Microhybrid

Single-shade	Omnichroma	Tokuyama, Tokyo, Japan	UDMA and TEGDMA	(79% wt and 68% vol) 250 nm spherical SiO_2_-ZrO_2_	Supra-nano filled

**Table 2:** Composition of immersion liquid used in the study.

Immersion media	Brand	Manufacturer	Composition
Artificial saliva	Biotène dry mouth relief mouthwash, fresh mint	GSK Group, Canada (105370AB)	Water, glyerin, xylitol, sorbitol, propylene glycol, poloxamer 407, sodium benzoate, hydroxyethylcellulose, methylparaben, propylparaben, flavor, sodium phosphate, and disodium phosphate

Black coffee	BOSS Coffee Iced long black	Frucor Suntory, Australia	Brewed coffee, water, coffee powder, coffee extract, flavors, and acidity regulator (500)

Red wine	Cleanskin Pinot Noir	Foodstuffs Own Brands Ltd., New Zealand	Sulphites and 12.5% alcohol volume

**Table 3:** Mean values of change in color of three different types of composite resins based on their immersion liquid and number of thermocycles.

∆E_00_ ± SE	Artificial saliva		Black coffee		Red wine	
	T_0_−T_1_	T_1_−T_2_	T_0_−T_1_	T_1_−T_2_	T_0_−T_1_	T_1_−T_2_
MS	2.26 ± 0.23	7.76 ± 1.20	11.89 ± 2.00	12.22 ± 1.46	17.15 ± 1.47	18.39 ± 1.06*⁣*^*∗*^
GS	3.11 ± 0.62	4.05 ± 0.47	8.49 ± 0.64*⁣*^*∗*^	9.00 ± 0.66*⁣*^*∗*^	7.88 ± 0.38*⁣*^*∗*^	9.27 ± 0.63*⁣*^*∗*^
SS	1.73 ± 0.27	2.29 ± 0.31	1.89 ± 0.09*⁣*^*∗*^	2.06 ± 0.08*⁣*^*∗*^	2.37 ±0.17*⁣*^*∗*^	1.06± 0.12*⁣*^*∗*^

*Note*: ∆E_00_: Change in color T_0_−T_1_: 5000 cycles, T_1_−T_2_ = additional 5000 cycles. *⁣*^*∗*^ = parametric data, otherwise it is nonparametric data.

Abbreviations: GS, group-shade; MS, multi-shade; SE, standard error; SS, single-shade.

**Table 4:** Mean values of relative translucency parameters of three different types of composite resins based on their immersion liquid and number of thermocycles.

RTP ± SE	Artificial saliva			Black coffee			Red wine		
	T_0_	T_1_	T_2_	T_0_	T_1_	T_2_	T_0_	T_1_	T_2_
MS	10.72 ± 0.54	11.15 ± 1.25	10.31 ± 1.03	10.72 ± 0.29	15.81 ± 2.62	14.78 ± 1.95	10.72 ± 0.54	*⁣* ^*∗*^16.67 ± 2.24	*⁣* ^*∗*^18.39 ± 1.79
GS	9.43 ± 0.21	10.06 ± 0.32	*⁣* ^*∗*^10.01 ± 0.31	9.43 ± 0.21	*⁣* ^*∗*^10.72 ± 2.97	*⁣* ^*∗*^11.36 ± 0.25	9.43 ± 0.21	*⁣* ^*∗*^11.88 ± 0.29	11.85 ± 0.36
SS	10.45 ± 0.11	*⁣* ^*∗*^10.29 ± 0.19	*⁣* ^*∗*^10.37 ± 0.24	10.45 ± 0.11	*⁣* ^*∗*^10.60 ± 0.23	*⁣* ^*∗*^10.48 ± 0.22	10.45 ± 0.12	*⁣* ^*∗*^10.44 ± 0.11	*⁣* ^*∗*^10.41 ± 0.16

*Note*: T_0_ = baseline, T_1_ = 5000 cycles, T_2_ = 10,000 cycles. = parametric data, otherwise it is nonparametric data.

**Table 5:** Mean values of surface roughness of three different types of composite resins based on their immersion liquid and number of thermocycles.

Sa ± SE	Artificial saliva			Black coffee			Red wine		
	T_0_	T_1_	T_2_	T_0_	T_1_	T_2_	T_0_	T_1_	T_2_
MS	0.12 ± 0.00	0.17 ± 0.01	0.16 ± 0.01	0.12 ± 0.01	0.11 ± 0.01	0.11 ± 0.01	0.12 ± 0.00	*⁣* ^*∗*^0.11 ± 0.01	*⁣* ^*∗*^0.13 ± 0.01
GS	0.18 ± 0.00	*⁣* ^*∗*^0.20 ± 0.01	0.23 ± 0.01	0.18 ± 0.00	0.21 ± 0.01	0.21 ± 0.01	0.18 ± 0.01	0.18 ± 0.01	0.15 ± 0.01
SS	0.10 ± 0.00	0.12 ± 0.00	0.14 ± 0.01	0.10 ± 0.00	0.13 ± 0.01	0.14 ± 0.01	0.10 ± 0.00	0.12 ± 0.01	0.12 ± 0.01

*Note*: T_0_ = baseline, T_1_ = 5000 cycles, T_2_ = 10,000 cycles. *⁣*^*∗*^ = parametric data, otherwise it is nonparametric data.

**Table 6:** Mean values of hardness of three different types of composite resins based on their immersion liquid and number of thermocycles.

VH ± SE	Artificial saliva			Black coffee			Red wine		
	T_0_	T_1_	T_2_	T_0_	T_1_	T_2_	T_0_	T_1_	T_2_
MS	99.22 ± 3.11	*⁣* ^*∗*^92.91 ± 1.33	91.00 ± 1.57	99.22 ± 3.11	*⁣* ^*∗*^ 84.34 ± 1.69	85.59 ± 1.99	99.22 ± 3.11	87.76 ± 1.31	*⁣* ^*∗*^87.76 ± 1.30
GS	*⁣* ^*∗*^63.56 ± 0.75	*⁣* ^*∗*^67.03 ± 0.92	*⁣* ^*∗*^62.11 ± 0.56	*⁣* ^*∗*^63.56 ± 0.75	*⁣* ^*∗*^65.02 ± 0.89	*⁣* ^*∗*^63.97 ± 1.07	*⁣* ^*∗*^63.56 ± 0.75	*⁣* ^*∗*^65.52 ± 0.70	*⁣* ^*∗*^61.73 ± 0.87
SS	*⁣* ^*∗*^64.93 ± 0.60	*⁣* ^*∗*^60.39 ± 0.60	*⁣* ^*∗*^60.53 ± 0.43	*⁣* ^*∗*^64.93 ± 0.60	*⁣* ^*∗*^60.92 ± 0.44	*⁣* ^*∗*^61.24 ± 0.35	*⁣* ^*∗*^64.93 ± 0.60	*⁣* ^*∗*^59.54 ± 0.57	*⁣* ^*∗*^56.97 ± 0.61

*Note*: T_0_ = baseline, T_1_ = 5000 cycles, T_2_ = 10,000 cycles. *⁣*^*∗*^ = parametric data, otherwise it is nonparametric data.

## Data Availability

The data that support the findings of this study are available from the corresponding author upon reasonable request.
